# Demographic and psychological predictors of alcohol use and misuse in autistic adults

**DOI:** 10.1177/1362361321992668

**Published:** 2021-07-07

**Authors:** Maya Bowri, Laura Hull, Carrie Allison, Paula Smith, Simon Baron-Cohen, Meng-Chuan Lai, William Mandy

**Affiliations:** 1 University College London, UK; 2 University of Cambridge, UK; 3 University of Toronto, Canada; 4 National Taiwan University Hospital and College of Medicine

**Keywords:** adult outcomes, adults, alcohol use, autism spectrum disorders, substance misuse

## Abstract

This study explored demographic and psychological predictors of alcohol use and misuse in a high-functioning, community sample of 237 autistic adults aged 18–75 (mean = 41.92 and standard deviation = 13.3) recruited in the United Kingdom. An online survey measured demographic information, autistic traits, depression, generalised anxiety, social anxiety, mental well-being, social camouflaging and alcohol use with the Alcohol Use Disorders Identification Test. The sample was divided into three groups (non-drinkers, non-hazardous drinkers and hazardous drinkers) and multinomial logistic regression models were used to investigate associations between alcohol use and demographic factors, autistic traits, mental health variables and social camouflaging. Our results demonstrated a U-shaped pattern among autistic adults, with non-drinkers and hazardous drinkers scoring significantly higher than non-hazardous drinkers on levels of autistic traits, depression, generalised anxiety and social anxiety. In multivariate analysis, autistic non-drinkers were less likely to be male (odds ratio = 0.44; 95% confidence interval = 0.22–0.87) and had more autistic traits (odds ratio = 2.50; 95% confidence interval = 1.19–5.28). Gender and level of autistic traits may be the most significant factors in predicting alcohol use in the autistic community.

**Lay abstract:**

Alcohol use and misuse are associated with a variety of negative physical, psychological and social consequences. The limited existing research on substance use including alcohol use in autistic adults has yielded mixed findings, with some studies concluding that autism reduces the likelihood of substance use and others suggesting that autism may increase an individual’s risk for substance misuse. This study investigated demographic and psychological predictors of alcohol use and misuse in a sample of 237 autistic adults. An online survey was used to obtain data on demographic information, autistic traits, depression, generalised anxiety, social anxiety, mental well-being, social camouflaging and alcohol use. The sample was divided into three groups (non-drinkers, non-hazardous drinkers and hazardous drinkers) in order to investigate associations between alcohol use and demographic factors, autistic traits, mental health variables and social camouflaging. Our results demonstrated a U-shaped pattern among autistic adults, with non-drinkers and hazardous drinkers scoring higher than non-hazardous drinkers on levels of autistic traits, depression, generalised anxiety and social anxiety. Autistic non-drinkers were less likely to be male and had more autistic traits. Gender and level of autistic traits may be the most significant factors in predicting alcohol use in the autistic community.

## Introduction

Alcohol consumption is common within general populations in the United Kingdom, Europe and North America, in part because it can help people feel relaxed and part of a social group (Abbey et al., 1993). In Western countries, alcohol use often starts in adolescence, when social norms are thought to be a major driver of alcohol consumption (Keyes et al., 2012). A large-scale national survey in 2017 found that 57% of people over the age of 16 years in the United Kingdom drank alcohol at least once in the week prior to being surveyed (Office for National Statistics [ONS], 2018). Whereas social affiliation has a significant role to play in alcohol *use*, mental health problems including depression and anxiety are likely be implicated in *misuse* in the general population, where alcohol could become a coping strategy for managing such problems (National Collaborating Centre for Mental Health [NCCMH], 2011). While moderate alcohol consumption is defined as having up to one drink per day for women and two drinks per day for men (Centers for Disease Control and Prevention, 2020), alcohol misuse is defined as alcohol consumption at a harmful or dependent level, and quantified as regularly drinking more than 14 units of alcohol per week (National Health Service, 2018). In the United Kingdom, 24% of adults in England and Scotland regularly drink above the government’s low-risk guidelines (Alcohol Change, 2020), despite alcohol misuse being a widely acknowledged public health concern and major cause for morbidity and mortality (NCCMH, 2011). Alcohol is implicated in a number of chronic physical health conditions and mental health difficulties, is associated with injury, accidents and adverse social outcomes including violence and relationship breakdown, and the economic burden of alcohol-related problems worldwide is substantial (NCCMH, 2011).

While alcohol use among the general population is widely documented and studied, little is known about alcohol use in autistic people, with previous studies having tended to investigate alcohol consumption by individuals with autism as part of a wider survey of substance use and physical health. Although limited, previous research on substance use (including alcohol use) among autistic adults suggests that the consequences of substance misuse may be exacerbated in autistic populations (Kronenberg et al., 2014). It is beneficial to study alcohol use/misuse as distinct from other substances in autistic adults given that compared to many other substances, alcohol is legal, easily accessible and widely used (Tinsley & Hendrickx, 2008), with reviews finding that alcohol is among the most used and misused substance among individuals with autism (Arnevik & Helverschou, 2016; Ressel et al., 2020).

Alcohol use has been historically considered rare among autistic adults (Butwicka et al., 2017). The assumed rarity of alcohol use among autistic adults may be a result of the social difficulties characteristic of autism; given that autistic adults may have less social contact compared to non-autistic adults, they may be less likely to use alcohol than non-autistic individuals (Clarke et al., 2016), and it has been hypothesised that core characteristics of autism may reduce exposure to social situations (e.g. parties), where alcohol is commonly consumed (Fortuna et al., 2015). Other characteristics not included in the diagnostic criteria for but frequently associated with autism may also explain the assumed rarity, including increased supervision over these individuals, a tendency towards low risk-taking and high harm-avoidance behaviour, and genetic differences in the biological reward system in this group (Santosh & Mijovic, 2006). These assumptions have been supported by previous studies which found significantly lower rates of alcohol consumption among autistic adults compared to both individuals with other psychiatric diagnoses and the general population (Croen et al., 2015; Santosh & Mijovic, 2006; Schapir et al., 2016).

More recently, however, contradictory findings have appeared to suggest that autism could increase an individual’s risk for substance *misuse* including alcohol misuse (Clarke et al., 2016). Substance misuse has been recognised as a co-occurring difficulty for some autistic adults, with one review reporting prevalence rates ranging from 0.7% to 36% (Arnevik & Helverschou, 2016) and another study finding problems related to substance use (including alcohol use disorder and somatic disease linked to alcohol misuse) in 19%–30% of autistic adults in clinical settings (Butwicka et al., 2017). Such findings are supported by the observation that higher levels of autistic traits are associated with higher risk for substance misuse in general population cohort studies (De Alwis et al., 2014; Lundström et al., 2011).

When considering the relationship between autistic characteristics and alcohol-related behaviour, it is important to distinguish between alcohol use and alcohol misuse. In a study of more than 3000 Australian adults from the general population, although those with high autistic trait scores were less likely to report alcohol consumption, they were at increased risk for developing alcohol dependence, leading the researchers to hypothesise that social difficulties may reduce the likelihood of onset of alcohol use in the autistic community, but upon initiation, progression to misuse and dependence may be accelerated in this group due to the repetitive behaviour associated with these conditions (De Alwis et al., 2014). Alcohol may be used by some autistic adults to manage difficulties associated with their autism, such as feelings of loneliness and isolation, social anxiety, poor sleep, rumination and boredom (Clarke et al., 2016). Depression and anxiety are common among autistic people (Lai & Baron-Cohen, 2015) and are associated with higher risk for alcohol misuse in the general population (Boschloo et al., 2013).

Demographic factors may be implicated in alcohol use among autistic people in similar or different ways to those in neurotypical adults. With respect to gender, in the general population, males are more likely to use alcohol than females (French et al., 2014), which has also been supported in two previous studies of autistic adults (Croen et al., 2015; Roy et al., 2015). Furthermore, in his autobiographical book, Matthew Tinsley suggests that for some autistic adults, alcohol may be used to cope with the demands and requirements of having a romantic partner or job (Tinsley & Hendrickx, 2008), which has been hypothesised elsewhere in the research literature (Roy et al., 2015). This idea contrasts with findings from the general population that college students who were single consumed more alcohol than those who were in a relationship (Pedersen et al., 2009), and a meta-analysis of longitudinal studies which found that being unmarried and unemployed were independently associated with increased alcohol consumption (Temple et al., 1991).

It has also been suggested that alcohol may be used by autistic adults to reduce anxiety in social situations, enhance social skills or mask social difficulties in order to achieve social inclusion (Clarke et al., 2016). ‘Camouflaging’ can be conceptualised as the manifestation of compensatory and masking strategies in an attempt to hide the features of one’s autism and is thought to represent a spectrum like autistic traits (Hull et al., 2017). Qualitative research found that reasons for camouflaging include to fit in and form relationships (Hull et al., 2017). Given that one common function of alcohol use in the general population is to fit in with others (Abbey et al., 1993), we hypothesise that camouflaging might be associated with alcohol use among autistic people, as those who camouflage more might be more likely to use alcohol as part of this process.

While alcohol use in autistic adults may be initially functional, serving to reduce distress and foster a sense of social inclusion, prolonged misuse may exacerbate mental health difficulties in this group (Clarke et al., 2016), and put the individual at risk of physical health problems. Lai and Baron-Cohen (2015) highlight the need for clinical assessment of autistic adults to establish whether substances are used to self-medicate, facilitate social interaction or have become an obsessive focus. However, currently little is known about variability in alcohol use among autistic adults, including what characteristics and mechanisms are associated with more or less drinking in this population. This makes screening for alcohol use and misuse in this population difficult, which is further constrained by the findings that autistic people often report barriers accessing healthcare due to difficulties with doctor–patient communication (Nicolaidis et al., 2013), and that healthcare professionals may be less likely to investigate alcohol use in autistic patients (Fortuna et al., 2015). Therefore, autistic adults may be less likely to receive alcohol misuse advice and support than non-autistic adults, maintaining these difficulties. Given the prevalence of alcohol use in Western countries and deinstitutionalisation resulting in more autistic individuals living in the community, it is imperative that we increase our understanding of alcohol use and misuse in this population.

The primary aim of our study was to investigate which demographic and psychological factors are associated with alcohol use and misuse among autistic adults, which to the authors’ knowledge is the first study to do so. First, we sought to investigate the relationship between alcohol use and demographic factors in autistic adults, specifically gender, relationship and occupational status. Second, given the mixed findings from previous studies regarding the effect of an autism diagnosis and autistic traits on alcohol use and misuse, our study sought to investigate the effect of autistic trait severity on alcohol use in this population. Third, while autobiographical and qualitative evidence suggests that co-occurring mental health variables including depression, generalised anxiety, social anxiety and low mental well-being are likely predictors of alcohol use in autistic adults (Clarke et al., 2016; Tinsley & Hendrickx, 2008), these factors have not been tested quantitatively. Finally, we sought to investigate the association between social camouflaging and alcohol use in autistic adults, as motivations for both camouflaging and alcohol use appear similar in qualitative research.

Our research questions were as follows:

*RQ1*. What are the proportions of participants who abstain from, use and misuse alcohol in our sample of autistic adults?
*RQ2*. What are the associations between gender, relationship and employment status and alcohol use and misuse in autistic adults?
*RQ3*. What is the association between autistic trait severity and alcohol use and misuse in autistic adults?
*RQ4*. What are the associations between levels of depression, generalised anxiety, social anxiety and mental well-being and alcohol use and misuse in autistic adults?
*RQ5*. What is the association between levels of social camouflaging and alcohol use and misuse in autistic adults?

## Methods

### Participants

Informed consent was sought from 320 participants. All participants were over the age of 18 years, fluent in English and self-reported an official autism diagnosis from a qualified healthcare professional. No independent verification of participants’ autism diagnoses was undertaken; however, participants were asked to detail the label of the diagnosis (e.g. autism, Asperger’s syndrome, autism spectrum disorder etc.), the age they were diagnosed and the type of healthcare professional who diagnosed them. Those who reported being self-diagnosed were automatically excluded from the study and did not complete any further questions. Owing to exclusion criteria (*n* = 14) and missing data (*n* = 55), 69 participants were excluded from the study, and the final sample consisted of 237 adults with an autism diagnosis (Supplemental Figure 1). Participants were recruited voluntarily via social media platforms (Twitter and Facebook), Internet forums (Autism Research Network and British Psychological Society Research Digest), the Cambridge Autism Research Database, and through Brighton Housing Trust, Dv8 Sussex vocational college and Autism Sussex charity (all based in South East England), and received no financial incentive to take part. Sensitivity power analysis using G*Power indicated our sample of 237 provided adequate (80%) power to detect an odds ratio of 1.5 or lower in logistic regression analysis, to identify significant predictors of being a non-drinker, non-hazardous or hazardous drinker.

### Procedure

The study was approved by the University College London Research Ethics Committee (ID no. 7475/002). The survey was hosted by Qualtrics. Informed consent was sought from participants after they had read a covering webpage by clicking ‘I agree’ in order to continue with the survey. The survey took approximately 30 minutes to complete. All responses were collected and stored anonymously and securely in accordance with the relevant data protection legislation. At the end of the survey, participants were provided with debriefing information including the researchers’ contact details and details for support organisations in the event that they had been affected by answering any of the questions. Due to the anonymous nature of responses, participants were not able to withdraw their responses once the survey was complete. All participants with missing data were removed from the study (*n* = 55), and all responses were screened for inconsistencies prior to analysis in order to reduce the likelihood of fraudsters (Teitcher et al., 2015).

### Measures

#### Demographic variables

Demographic information was obtained, including participants’ autism diagnoses/severity (e.g. Asperger’s syndrome, high-functioning autism, autism spectrum disorder/condition, autistic disorder and pervasive developmental disorder–not otherwise specified (PDD-NOS)) and co-occurring psychiatric/medical conditions, age, gender, nationality, relationship status, occupation and education level.

#### Autistic traits

Autistic traits were measured using the Broad Autism Phenotype Questionnaire (BAPQ; Hurley et al., 2007). While initially developed to measure autistic-like traits in relatives of autistic people, the BAPQ is a good measure of variability in autistic traits of autistic individuals (Nishiyama et al., 2014). The BAPQ has 36 items and three subscales: social deficits, stereotyped repetitive behaviours and social language deficits. Minimum score is 0 and the maximum is 6 when scores are averaged across the total questionnaire. Scores of 3.55 and 3.17 are taken as the cut-off values for males and females (Sasson et al., 2013), respectively. The BAPQ has good sensitivity (Sasson et al., 2013) and specificity (Hurley et al., 2007). Internal consistency in this sample was high (*a* = 0.92).

#### Mental health variables

Depression was measured using the Patient Health Questionnaire (PHQ-9; Kroenke et al., 2001) which has nine items corresponding to the *Diagnostic and Statistical Manual of Mental Disorders* (4th ed.; DSM-IV) diagnostic criteria for depression. Scores of 5, 10, 15 and 20 represent cut points for mild, moderate, moderately severe and severe depression, respectively. The PHQ-9 correlates highly with a number of quality of life, functional status and healthcare usage measures (Kroenke & Spitzer, 2002) and has excellent internal and external reliability (Kroenke et al., 2001). Internal consistency in this sample was good (*ɑ* = 0.89).

Generalised anxiety was measured using the seven-item Generalised Anxiety Disorder Questionnaire (GAD-7; Spitzer et al., 2006) used to screen and dimensionally measure generalised anxiety disorder. Scores of 5, 10 and 15 are indicative of mild, moderate and severe generalised anxiety (Spitzer et al., 2006), respectively. The GAD-7 has good convergent validity with worry, anxiety, depression and stress (Kertz et al., 2013) and has excellent internal and external reliability (Spitzer et al., 2006). Internal consistency in this sample was high (*ɑ* = 0.92).

Social anxiety was measured using the Liebowitz Social Anxiety Scale (LSAS; Liebowitz, 1987), which measures fear and associated avoidance in 24 situations. Total scores are interpreted as follows: 50–65 ‘moderate social phobia’, 65–80 ‘marked social phobia’, 80–95 ‘severe social phobia’ and >95 ‘very severe social phobia’. The LSAS correlates significantly with other common measures for social anxiety and avoidance (Heimberg et al., 1999) and has high internal consistency (Fresco et al., 2001). Internal consistency in this sample was high (*ɑ* = 0.96).

Mental well-being was measured using the Warwick–Edinburgh Mental Well-being Scale (WEMWBS; Tennant et al., 2007), which has 14 items concerning mood, relationships and functioning. Higher scores indicate higher levels of mental well-being. The WEMWBS has excellent internal and external reliability, and correlates highly with other measures for mental health and well-being (Tennant et al., 2007). Internal consistency in this sample was good (*ɑ* = 0.89).

#### Social camouflaging

Camouflaging was measured using the 25-item Camouflaging Autistic Traits Questionnaire (CAT-Q; Hull et al., 2019). The CAT-Q measures an individual’s use of camouflaging strategies to compensate for and/or mask their autistic characteristics in social interactions. Higher scores indicate more camouflaging. Preliminary validation in autistic and general populations has found good levels of internal consistency for all three factors (compensation: *a* = 0.92; masking: *a* = 0.86; assimilation: *a* = 0.93). Internal consistency in this sample was excellent (*ɑ* = 0.91).

#### Alcohol use

Alcohol use was measured using the Alcohol Use Disorders Identification Test (AUDIT; Saunders et al., 1993), a widely used screening instrument for harmful or hazardous alcohol consumption originally developed for use in primary healthcare settings. The AUDIT has a high level of test–retest reliability and correlates highly with other common alcohol screening instruments (Babor et al., 2001). The AUDIT has 10 items that cover the domains of alcohol consumption, drinking behaviour and alcohol-related problems. The first question measures frequency of alcohol consumption, and participants who report abstinence from drinking alcohol are instructed to skip to the end of the questionnaire. Scores of 8 or more indicate harmful alcohol use and possible dependence (Babor et al., 2001).

### Analyses

Analyses were conducted using IBM SPSS Statistics version 24. Chi-square tests were used for comparisons between the final sample and participants excluded on the basis of exclusion criteria or missing data. Distributions for all study variables were examined for skewness and kurtosis.

One-way analysis of variance (ANOVA) was performed to test for gender differences in depression, generalised anxiety, social anxiety, mental well-being, autistic traits and social camouflaging. Mann–Whitney tests were used to examine the association between relationship status and AUDIT scores. Kruskal–Wallis tests were used to examine the association between alcohol use and gender and employment status. Spearman’s correlation was used to examine associations between alcohol use, autistic traits, depression, generalised anxiety, social anxiety, mental well-being and social camouflaging.

The sample was divided into three groups based on standard classification AUDIT scores (non-drinkers (0), non-hazardous drinkers (1–8) and hazardous drinkers (⩾8); Babor et al., 2001) and unadjusted multinomial logistic regression models investigated associations between alcohol consumption and independent variables. Relationship status categories were collapsed according to whether participants were in a relationship (i.e. they reported being ‘in a relationship’, ‘engaged’ or ‘married’) versus not in a relationship (‘single’, ‘divorced’ or ‘widowed’). An adjusted multivariate multinomial logistic regression model then investigated the combined effect of all significant predictors from the unadjusted models. ‘Non-hazardous drinkers’ was used as a reference category for all multinomial logistic regression analyses. Critical level for statistical significance for all analyses was set at 0.05.

## Results

### Analysis of excluded cases

Chi-square tests found that participants excluded owing to exclusion criteria or missing data (*N* = 69) did not differ from the final sample (*N* = 237) on gender, relationship status, education level, employment or any other independent variable (all *p*s > 0.05).

### Descriptive data

Demographic and mental health characteristics of the sample are presented in Tables 1 and 2. Participants had an average age of 41.92 years, with the majority being female (*N* = 139, 58.6%). The majority of participants (*N* = 186, 78.5%) identified themselves as having ‘Asperger’s syndrome’ or ‘high-functioning autism’, and the average age of diagnosis was 35.98. Most participants (*N* = 167, 70.5%) reported that they had additional diagnoses/disabilities other than their autism, and over half of the sample reported co-occurring anxiety and depression disorders (*N* = 134, 56.5% and *N* = 128, 54.0%).

**Table 1.** Demographic characteristics.

Characteristic	*N* = 237
Age, mean (SD)	41.92 (13.3)
Age, categories, *N* (%)	
18–24	23 (9.7)
25–34	60 (25.3)
35–44	49 (20.7)
45–54	56 (23.6)
55–64	32 (13.5)
65–74	11 (4.6)
75 and over	5 (2.1)
Gender, *N* (%)	
Female	139 (58.6)
Male	83 (35.0)
Other	13 (5.5)
Undisclosed	2 (0.8)
Relationship status, *N* (%)	
Single	104 (43.9)
In a relationship	46 (19.4)
Engaged	3 (1.3)
Married	68 (28.7)
Divorced	14 (5.9)
Widowed	1 (0.4)
Undisclosed	1 (0.4)
Occupation status, *N* (%)	
Employed full-time	75 (31.6)
Employed part-time	35 (14.8)
Volunteer	7 (3.0)
Student	33 (13.9)
Unemployed	30 (12.7)
Homemaker	12 (5.1)
Retired	19 (8.0)
Other	24 (10.1)
Undisclosed	2 (0.8)
Highest education level, *N* (%)	
Secondary school	47 (19.8)
Apprenticeship/college	41 (17.3)
Undergraduate degree	72 (30.4)
Postgraduate degree	76 (31.6)
Undisclosed	2 (0.8)
Nationality, *N* (%)	
European (including the United Kingdom and Ireland)	169 (71.3)
North American (Canada and the United States)	44 (18.6)
South American (Brazil)	2 (0.8)
Asian (Japan and Singapore)	2 (0.8)
Australasian (Australia and New Zealand)	11 (4.6)
Dual/mixed nationality	6 (2.5)
Undisclosed	3 (1.3)
ASC diagnosis, *N* (%)	
Asperger’s syndrome; high-functioning autism	186 (78.5)
Autism spectrum disorder/condition; autism	41 (17.3)
Classic autism; autistic disorder	3 (1.3)
PDD-NOS	7 (3.0)
Age at ASC diagnosis, mean (SD)	35.98 (13.4)

SD: standard deviation; ASC: autism spectrum condition; PDD-NOS: pervasive developmental disorder–not otherwise specified.

**Table 2.** Mental health characteristics and gender difference statistics.

	Total (*N* = 237)	Males (*N* = 83)	Females (*N* = 139)	Significance testing statistics
Co-occurring diagnoses/disabilities, *N* (%)				
Yes	167 (70.5)			
No	59 (24.9)			
Undisclosed	11 (4.6)			
Co-occurring diagnoses/disabilities, *N* (%)				
Anxiety	134 (56.5)			
Depression	128 (54.0)			
ADHD	25 (10.5)			
ID/LD	11 (4.6)			
Other	79 (33.3)			
AUDIT score, mean (SD)	3.63 (4.99)	4.57 (5.70)	3.16 (4.61)	M > F (*p* = 0.021, *r* = 0.18)
PHQ-9 score, mean (SD)	11.83 (6.90)	10.67 (7.36)	12.25 (6.50)	NS (*p* = 0.095, *r* = 0.13)
GAD-7 score, mean (SD)	9.78 (6.15)	8.72 (6.18)	10.24 (6.07)	NS (*p* = 0.118, *r* = 0.13)
LSAS score, mean (SD)	84.31 (29.57)	77.29 (31.89)	89.06 (28.16)	F > M (*p* = 0.012, *d* = 0.40)
WEMWBS score, mean (SD)	38.13 (8.80)	38.59 (9.33)	37.73 (8.26)	NS (*p* = 0.682, *r* = 0.06)
BAPQ score, mean (SD)	4.37 (0.67)	4.28 (0.77)	4.44 (0.63)	NS (*p* = 0.166, *r* = 0.120)
CAT-Q score, mean (SD)	120.16 (24.24)	111.16 (25.36)	125.37 (22.69)	F > M (*p* = 0.001, *d* = 0.60)

ADHD: attention deficit hyperactivity disorder; ID: intellectual disability; LD: learning difficulty; AUDIT: Alcohol Use Disorders Identification Test; PHQ: Patient Health Questionnaire; GAD: Generalised Anxiety Disorder Questionnaire; LSAS: Liebowitz Social Anxiety Scale; WEMWBS: Warwick–Edinburgh Mental Well-being Scale; BAPQ: Broad Autism Phenotype Questionnaire; CAT-Q: Camouflaging Autistic Traits Questionnaire; NS: not significant.

AUDIT (alcohol use); PHQ-9 (depression); GAD-7 (generalised anxiety); LSAS (social anxiety); WEMWBS (well-being); BAPQ (autistic traits); CAT-Q (camouflaging).

### Distributions of key variables

Scores on the AUDIT were positively skewed. Visual inspection of all other variables, skewness and kurtosis values and consideration of the sample size (Field, 2013) indicated that these variables satisfied assumption of normality. Assumption of homogeneity of variance was met for all parametric tests.

### Proportions of non-drinkers, non-hazardous drinkers and hazardous drinkers

Seventy-one participants (30.0%) reported abstaining from alcohol. One hundred and thirty participants (54.8%) reported drinking occasionally to moderately, while 36 participants’ (15.2%) scores were indicative of hazardous drinking.

### Bivariate linear associations

Spearman’s correlations between study variables are presented in Table 3. Scores on the AUDIT were not significantly associated with any of the independent variables, which may reflect the relatively high proportion of non-drinkers in our sample who scored zero on this measure, or non-linear relationships between these variables. Autistic traits were positively correlated with depression, generalised anxiety, social anxiety and camouflaging, and negatively correlated with well-being. Camouflaging was positively correlated with depression, generalised anxiety and social anxiety.

**Table 3.** Spearman’s correlations between study variables (*N* = 237).

	AUDIT	BAPQ	PHQ-9	GAD-7	LSAS	WEMWBS	CAT-Q
AUDIT							
BAPQ	−0.103						
PHQ-9	0.011	0.292**					
GAD-7	0.002	0.318**	0.747**				
LSAS	−0.043	0.562**	0.417**	0.423**			
WEMWBS	0.029	−0.455**	−0.721**	−0.598**	−0.370**		
CAT-Q	0.046	0.257**	0.221**	0.288**	0.373**	−0.122	

AUDIT: Alcohol Use Disorders Identification Test; PHQ: Patient Health Questionnaire; GAD: Generalised Anxiety Disorder Questionnaire; LSAS: Liebowitz Social Anxiety Scale; WEMWBS: Warwick–Edinburgh Mental Well-being Scale; BAPQ: Broad Autism Phenotype Questionnaire; CAT-Q: Camouflaging Autistic Traits Questionnaire.

AUDIT: alcohol use; BAPQ: autistic traits; PHQ-9: depression; GAD-7: generalised anxiety; LSAS: social anxiety; WEMWBS: well-being; CAT-Q: camouflaging.

** *p* < 0.01.

## Univariate analyses

### Associations between demographic variables and alcohol use/misuse

There was a significant main effect of gender on scores on the AUDIT, *H*(2) = 7.45, *p* = 0.024. Males had higher AUDIT scores than females (Table 2). Multinomial logistic regression analysis found being male to be associated with lower odds of being a non-drinker (Table 4).

**Table 4.** Univariate models for associations between alcohol consumption and independent variables (*N* = 237).

	Non-drinkers (vs non-hazardous drinkers^a^)		Hazardous drinkers (vs non-hazardous drinkers^a^)	
	*β* (SE)	Crude OR (95% CI)	*β* (SE)	Crude OR (95% CI)
Male vs female/other^b^	−0.893 (0.335)	0.41 (0.21–0.79)**	−0.229 (0.390)	0.80 (0.37–1.71)
No partner vs partner^b^	0.519 (0.301)	1.68 (0.93–3.03)	−0.749 (0.404)	0.47 (0.21–1.04)
Autistic traits	0.023 (0.007)	1.02 (1.00–1.04)**	0.020 (0.009)	1.02 (1.00–1.04)*
Depression	0.045 (0.022)	1.05 (1.00–1.09)*	0.090 (0.028)	1.09 (1.04–1.16)**
Anxiety	0.061 (0.025)	1.06 (1.01–1.12)*	0.093 (0.032)	1.10 (1.03–1.17)**
Social anxiety	0.013 (0.005)	1.01 (1.00–1.02)*	0.017 (0.007)	1.02 (1.00–1.03)*
Mental well-being	−0.003 (0.017)	0.97 (0.94–1.00)	−0.054 (0.022)	0.95 (0.91–0.99)*
Camouflaging	0.004 (0.006)	1.00 (0.99–1.02)	0.014 (0.008)	1.01 (1.00–1.03)

SE: standard error; OR: odds ratio; CI: confidence interval.

a Reference category for dependent variables.

b Reference category for independent variables.

* *p* < 0.05; **p < 0.01.

Participants who were single, divorced or widowed scored lower on the AUDIT than participants who were in a relationship, engaged or married, *U* = 8147.00, *z* *=* 2.30, *p* *=* 0.021, *r* = 0.15. Relationship status was not associated with alcohol consumption in multinomial logistic regression analysis (Table 4). The association between employment status and scores on the AUDIT was non-significant, and therefore, employment status was excluded from further analysis. No significant differences in AUDIT scores were found between participants with and without additional diagnoses/disabilities, and these variables were excluded from further analyses.

### Association between autistic trait severity and alcohol use/misuse

Higher autistic traits were associated with both being a non-drinker and a hazardous drinker compared to the reference category of being a non-hazardous drinker (Table 4).

### Associations between mental health variables and alcohol use/misuse

Higher levels of depression, generalised anxiety and social anxiety were associated with being both a non-drinker and a hazardous drinker (Table 4). Lower levels of mental well-being were associated with being a hazardous drinker.

### Association between social camouflaging and alcohol use/misuse

Social camouflaging was not associated with alcohol consumption (Table 4). Mean values, standard deviations and 95% confidence intervals for continuous study variables are presented in Table 5. Mean depression, generalised anxiety, social anxiety, well-being and autistic trait scores for the three groups are further displayed in Supplemental Figure 2(a) to (e).

**Table 5.** Mean scores, standard deviations and 95% confidence intervals for psychological variables for non-drinkers, non-hazardous drinkers and hazardous drinkers (*N* = 237).

Measure (range)	Non-drinkers (ND)	Non-hazardous drinkers (NHD)	Hazardous drinkers (HD)
BAPQ (1.89–6.00)	4.57 (0.59); 4.43–4.71	4.23 (0.69); 4.11–4.35	4.52 (0.66); 4.30–4.74
PHQ-9 (0–27)	12.61 (7.33); 10.90–14.32	10.58 (6.55); 9.45–11.71	14.78 (6.35); 12.71–16.85
GAD-7 (0–21)	10.82 (6.54); 9.30–12.34	8.60 (5.82); 7.60–9.60	12.03 (5.71); 10.16–13.90
LSAS (0–139)	90.04 (31.15); 82.80–97.28	78.82 (28.14); 73.98–83.66	90.56 (27.98); 81.42–99.70
WEMWBS (14–70)	37.01 (9.12); 34.89–39.13	39.48 (8.60); 38.00–40.96	35.44 (8.13); 32.78–38.10
CAT-Q (25–175)	120.52 (23.24); 115.12–125.92	118.29 (24.58); 114.06–122.52	126.19 (24.61); 118.15–134.23

AUDIT: Alcohol Use Disorders Identification Test; PHQ: Patient Health Questionnaire; GAD: Generalised Anxiety Disorder Questionnaire; LSAS: Liebowitz Social Anxiety Scale; WEMWBS: Warwick–Edinburgh Mental Well-being Scale; BAPQ: Broad Autism Phenotype Questionnaire; CAT-Q: Camouflaging Autistic Traits Questionnaire.

BAPQ: autism traits; PHQ-9: depression; GAD-7: generalised anxiety; LSAS: social anxiety; WEMWBS: well-being; CAT-Q: camouflaging.

## Multivariate analysis

The adjusted multinomial regression model for associations between alcohol consumption and significant predictors from the unadjusted models is presented in Table 6. The model was significant, *χ*^2^ (14) = 34.44, *p* < 0.01, and the Pearson, *χ*^2^ (458) = 470.21, *p* = 0.337, and deviance, *χ*^2^ (458) = 428.55, *p* = 0.835; chi-square statistics showed the model was a good fit for the data. The model correctly classified 56.1% of cases and explained 16% of the variance in alcohol consumption. Being male continued to be associated with reduced odds of being a non-drinker, while autistic traits continued to be associated with increased odds of being a non-drinker.

**Table 6.** Multivariate model for associations between alcohol consumption and independent variables (*N* = 237).

	Non-drinkers (vs non-hazardous drinkers^a^)		Hazardous drinkers (vs non-hazardous drinkers^a^)	
	*β* (SE)	Adjusted OR (95% CI)	*β* (SE)	Adjusted OR (95% CI)
Male vs female/other^b^	−0.830 (0.354)	0.44 (0.22–0.87)*	0.035 (0.415)	1.04 (0.46–2.34)
Depression	−0.003 (0.040)	1.00 (0.92–1.08)	0.065 (0.048)	1.07 (0.97–1.17)
Anxiety	0.040 (0.038)	1.04 (0.97–1.12)	0.020 (0.049)	1.02 (0.93–1.12)
Social anxiety	0.002 (0.007)	1.00 (0.99–1.02)	0.002 (0.009)	1.00 (0.98–1.02)
Mental well-being	0.007 (0.027)	1.01 (0.96–1.06)	0.010 (0.035)	1.01 (0.94–1.08)
Autistic traits	0.918 (0.380)	2.50 (1.19–5.28)*	–0.072 (0.489)	0.93 (0.36–2.42)

SE: standard error; OR: odds ratio; CI: confidence interval.

*R*^2^ = 0.14 (Cox and Snell) and *R*^2^ = 0.16 (Nagelkerke). Model *χ*^2^(14) = 34.44, *p* < 0.01.

a Reference category for dependent variables.

b Reference category for independent variables.

* *p* < 0.05.

## Discussion

This study explored demographic and psychological predictors of alcohol use and misuse in a sample of autistic adults. Thirty percent of our sample reported abstaining from alcohol, which is higher than the 20.4% of adults who reported teetotalism in a 2017 general population survey based in the United Kingdom (ONS, 2018). 55% of our sample reported consuming alcohol occasionally to moderately, while 15.2% were classified as hazardous drinkers. The proportion of hazardous drinkers in our study is comparable to the proportion of hazardous drinkers (14%) observed a general population study in Sweden which used the AUDIT (Lundin et al., 2015). These findings suggest that autistic adults may be more likely to abstain from alcohol than their neurotypical counterparts, and equally likely to misuse alcohol when compared with non-autistic individuals, although comparison data from the United Kingdom and Sweden may not be representative of teetotalism and hazardous drinking rates more globally and thus should be interpreted cautiously against our sample. To draw more accurate conclusions, it is essential to compare rates of alcohol use and misuse within large, population-based samples of autistic and non-autistic adults. A recent longitudinal twin study in Sweden reported a lower prevalence of risk drinking in autistic adolescents and young adults at ages 15, 18 and 24 years compared to their twins without autism (Kaltenegger et al., 2021). However, the study found a continual increase in the prevalence of risk drinking among autistic individuals with age (rising to 13% at age 24 years), and it is therefore important to acknowledge the higher mean age of our sample, potential for the prevalence of hazardous drinking among autistic adults to increase across the lifespan and to be comparable to rates from the general population.

Scores for independent variables across the three groups demonstrated a U-shaped pattern, with hazardous drinkers and non-drinkers scoring higher on autistic traits, depression, generalised anxiety and social anxiety than non-hazardous drinkers. For mental well-being, an equivalent pattern prevailed, with non-hazardous drinkers reporting the highest well-being, followed by non-drinkers, with hazardous drinkers reporting the lowest levels of well-being. Taken together, these findings suggest that non-hazardous consumption of alcohol is associated with better mental health for autistic adults relative to teetotalism, which supports findings from previous qualitative research (Kronenberg et al., 2015; Lalanne et al., 2015), while hazardous alcohol consumption is associated with poorer mental health in this group.

Unadjusted multinomial logistic regression models using non-hazardous drinkers as a reference category found being male to reduce the likelihood of abstaining from alcohol, while autistic traits, depression, generalised anxiety and social anxiety were associated with an increased likelihood of teetotalism. Autistic traits, depression, generalised anxiety and social anxiety were associated with increased likelihood of hazardous alcohol consumption, while mental well-being reduced this risk. No significant relationship between alcohol use and camouflaging was found, suggesting that although camouflaging is related to mental health problems generally (Hull et al., 2019; Lai et al., 2017), it did not seem to directly increase likelihood of alcohol use or misuse in this sample.

When significant predictors from unadjusted models were combined in the adjusted model, only gender and autistic traits were significant when controlling for the other variables. Being male continued to be associated with a reduced likelihood of teetotalism, while autistic traits were associated with a two-and-a-half-fold increase in the likelihood of abstaining from alcohol. These findings suggest that an individual’s gender and level of autistic traits may be the most significant predictors of alcohol use among autistic adults. These findings provide support for existing research which has found autistic males to be more likely to drink alcohol than autistic females, following the same pattern as the general population.

It is not clear from this study which mechanisms are behind the association between autistic traits and alcohol consumption, which was corroborated in a general population study (De Alwis et al., 2014). One potential explanation is that higher levels of autistic traits are associated with greater social deficits, leading to reduced motivation to enter social situations where alcohol is consumed and therefore a lower tendency to access alcohol and/or experience social expectation to drink. Another possibility is that higher autistic traits are associated with greater cognitive rigidity, which may result in ‘black-and-white thinking’ with regard to the acceptability of drinking, leading to lower alcohol consumption. There were no unique predictors of hazardous drinking in the adjusted model, which could be explained by the relatively low proportion of hazardous drinkers in our sample and consequential lack of power. However, for a substantial proportion of autistic people, particularly males and/or those individuals with lower levels of autistic traits, alcohol in moderation may be a means to self-medicate co-occurring mental health difficulties, thus accounting for the seemingly ‘optimal’ level of alcohol use observed in our study. Autistic adults have previously described using alcohol to self-medicate both co-occurring mental health difficulties, and some of the sensory and social difficulties associated with being autistic in a neurotypical world (Tinsley & Hendrickx, 2008). This has important implications for clinical practice, as such individuals might be less likely to use alcohol if they were able to develop alternative coping strategies for navigating the world and their diagnosis.

This is the first study to quantitatively investigate demographic and psychological predictors of alcohol use and misuse by autistic people. The sample was relatively large and included a high proportion of females who are typically under-represented in autism research (Hull et al., 2017). The use of the AUDIT is a major strength of our study since previous research on alcohol use in autistic adults has been limited by a lack of standardised measures, with several studies having employed only a single item to measure alcohol use/misuse (Ressel et al., 2020). Nevertheless, the limitations of this study warrant consideration. Since the study was cross-sectional, it is not possible to draw conclusions regarding directionality or causation. It is not clear whether autistic adults misuse alcohol to self-medicate or whether hazardous alcohol use impacts negatively on mental health, or whether the relationship between alcohol use and mental health problems is bidirectional. Similarly, it is not clear whether non-hazardous consumption of alcohol acts as a protective factor against depression and anxiety or whether individuals with lower depression and anxiety are more likely to consume alcohol at non-hazardous levels. Other factors which were not measured in this study but may account for the findings observed include reduced alcohol consumption or teetotalism in participants who might have been taking medications for depression and anxiety, variation in access to alcohol, coping strategies (Kronenberg et al., 2015), family history of substance use disorder (Sizoo et al., 2010) and beliefs that alcohol is an effective way to manage distress and enhance social skills (Clarke et al., 2016). Our findings should therefore be interpreted cautiously, and future research should aim to elucidate these mechanisms with the inclusion of additional measures.

Most participants identified themselves as having ‘Asperger syndrome’ or ‘high-functioning autism’, which may coincide with the relatively late average age of diagnosis observed in our sample (Happe et al., 2016). It is likely that an even higher proportion of our sample was high functioning given the publication of the *Diagnostic and Statistical Manual of Mental Disorders* (5th ed.; DSM-5) in 2013 and single label of autism spectrum disorder replacing previous subtypes. Our sample is not representative of the broad spectrum and the nature of the study largely excluded individuals with lower intellectual ability who may have been unable to complete the survey. Future research should aim to recruit a wider range of individuals from across the spectrum using more accessible measures. The reliance on self-reported autism diagnoses which were not independently verified is another limitation of our study. In conclusion, this study provides support for the roles of depression, generalised anxiety, social anxiety and mental well-being in alcohol use and misuse in autistic adults and enhances this knowledge by highlighting the importance of gender and autistic traits in alcohol use in this group.

### Recommendations

Based on our findings, the authors recommend that screening for alcohol use should form part of routine physical healthcare and mental health assessments for autistic adults given that a significant proportion of these individuals may be misusing alcohol. An individualised approach should then be taken to explore the unique combination of factors involved in alcohol misuse for those identified to be at-risk, in order to pave the way for tailored treatment and support. Clinicians should be aware that autistic adults may be using alcohol for both similar (e.g. self-medication of mental health problems) and different (e.g. to cope with sensory overstimulation during socialisation) reasons to non-autistic adults. Alternative coping strategies should be discussed and trialled to minimise hazardous alcohol use in this population. Research in this area continues to remain sparse (Adhia et al., 2020), and we call for future researchers in the field of autism and/or alcohol to build on our findings.

## Supplemental Material

sj-docx-1-aut-10.1177_1362361321992668 – Supplemental material for Demographic and psychological predictors of alcohol use and misuse in autistic adults Click here for additional data file. Supplemental material, sj-docx-1-aut-10.1177_1362361321992668 for Demographic and psychological predictors of alcohol use and misuse in autistic adults by Maya Bowri, Laura Hull, Carrie Allison, Paula Smith, Simon Baron-Cohen, Meng-Chuan Lai and William Mandy in Autism

sj-docx-2-aut-10.1177_1362361321992668 – Supplemental material for Demographic and psychological predictors of alcohol use and misuse in autistic adults Click here for additional data file. Supplemental material, sj-docx-2-aut-10.1177_1362361321992668 for Demographic and psychological predictors of alcohol use and misuse in autistic adults by Maya Bowri, Laura Hull, Carrie Allison, Paula Smith, Simon Baron-Cohen, Meng-Chuan Lai and William Mandy in Autism
