# Supplementary material for: Demographic and psychological predictors of alcohol use and misuse in autistic adults
Source: Autism. 2021 Jul 7;25(5):1469–80. doi: 10.1177/1362361321992668 (PMC8264632; doi:10.1177/1362361321992668)
Supplement: sj-docx-1-aut-10.1177_1362361321992668 – Supplemental material for Demographic and psychological predictors of alcohol use and misuse in autistic adults [file sj-docx-1-aut-10.1177_1362361321992668.docx]

Supplementary Figure 1. *Flow diagram of sample selection*

Consented to participate in study; assessed for eligibility (*N* = 320)

Did not meet inclusion criteria

(*N* = 14)

Confirmed eligible

(*N* = 306)

Missing full set of demographic variables (*N* = 3)

Full set of values for demographic variables (*N* = 303)

Missing full set of CAT-Q values (*N* = 17)

Full set of values for CAT-Q

(*N* = 286)

Missing full set of BAPQ values (*N* = 13)

Full set of values for BAPQ

(*N* = 273)

Missing full set of LSAS values (*N* = 33)

Full set of values for LSAS, WEMWBS & AUDIT

(*N* = 240)

Missing full set of PHQ-9 values (*N* = 1)

Full set of values for PHQ-9 (*N* = 239)

Missing full set of GAD-7 values (*N* = 2)

Full set of values for GAD-7; final sample (*N* = 237)
