# Supplementary material for: Demographic and psychological predictors of alcohol use and misuse in autistic adults
Source: Autism. 2021 Jul 7;25(5):1469–80. doi: 10.1177/1362361321992668 (PMC8264632; doi:10.1177/1362361321992668)
Supplement: sj-docx-2-aut-10.1177_1362361321992668 – Supplemental material for Demographic and psychological predictors of alcohol use and misuse in autistic adults [file sj-docx-2-aut-10.1177_1362361321992668.docx]

Supplementary Figures 2a-e. *Mean a) depression; b) generalised anxiety; c) social anxiety; d) autism trait; and e) mental wellbeing scores for non-drinkers, non-hazardous drinkers and hazardous drinkers. Error bars represent standard deviations.*

**
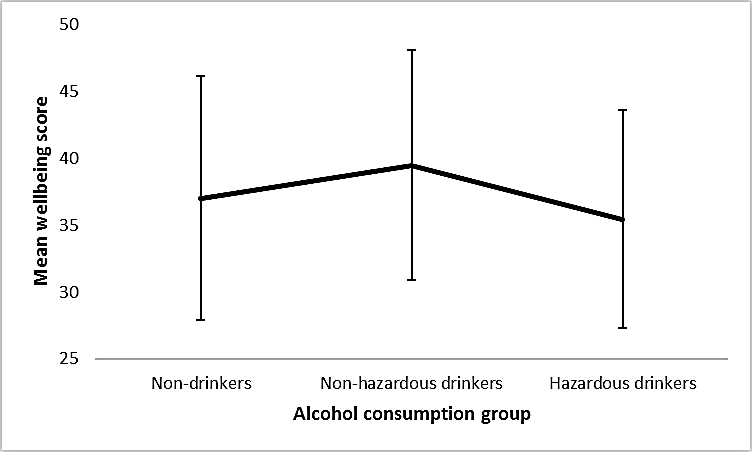

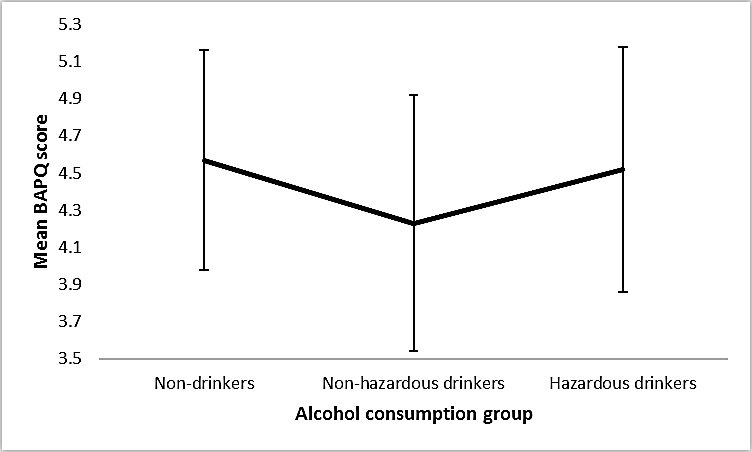
**
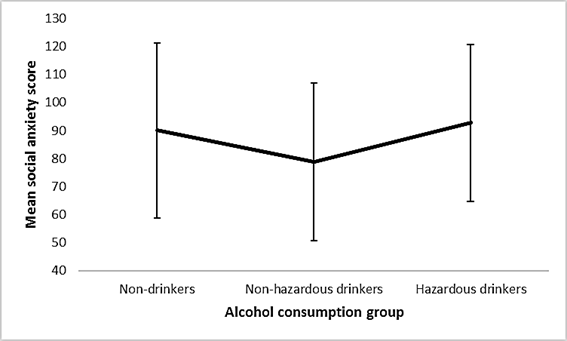
**
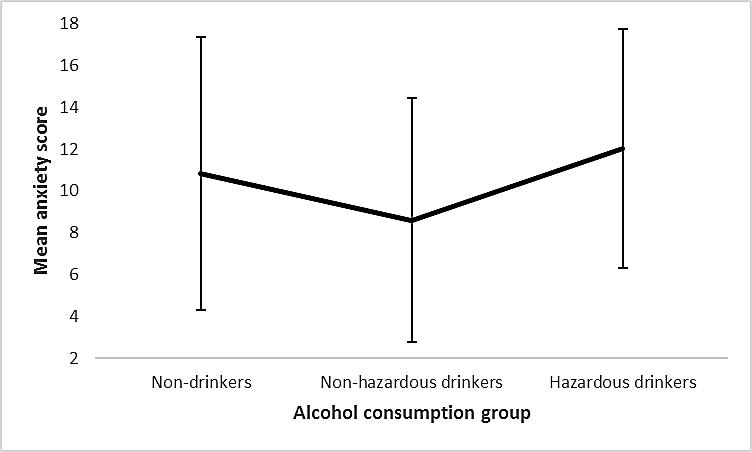
**
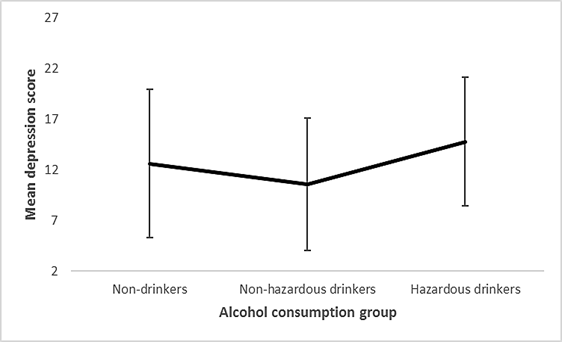


Fig. 2e

Fig. 2d

Fig. 2c

Fig. 2b

Fig. 2a
